# Supplementary material for: A Global Screen for Assembly State Changes of the Mitotic Proteome by SEC-SWATH-MS
Source: Cell Syst. 2020 Feb 26;10(2):133–155.e6. doi: 10.1016/j.cels.2020.01.001 (PMC7042714; doi:10.1016/j.cels.2020.01.001)

Q9BQA1 | MEP50_HUMAN | WDR77 MEP50 WD45 HKMT1069 Nbla10071
Monomer MW [kDa]: 36.724 Monomer expected elution fraction: 49

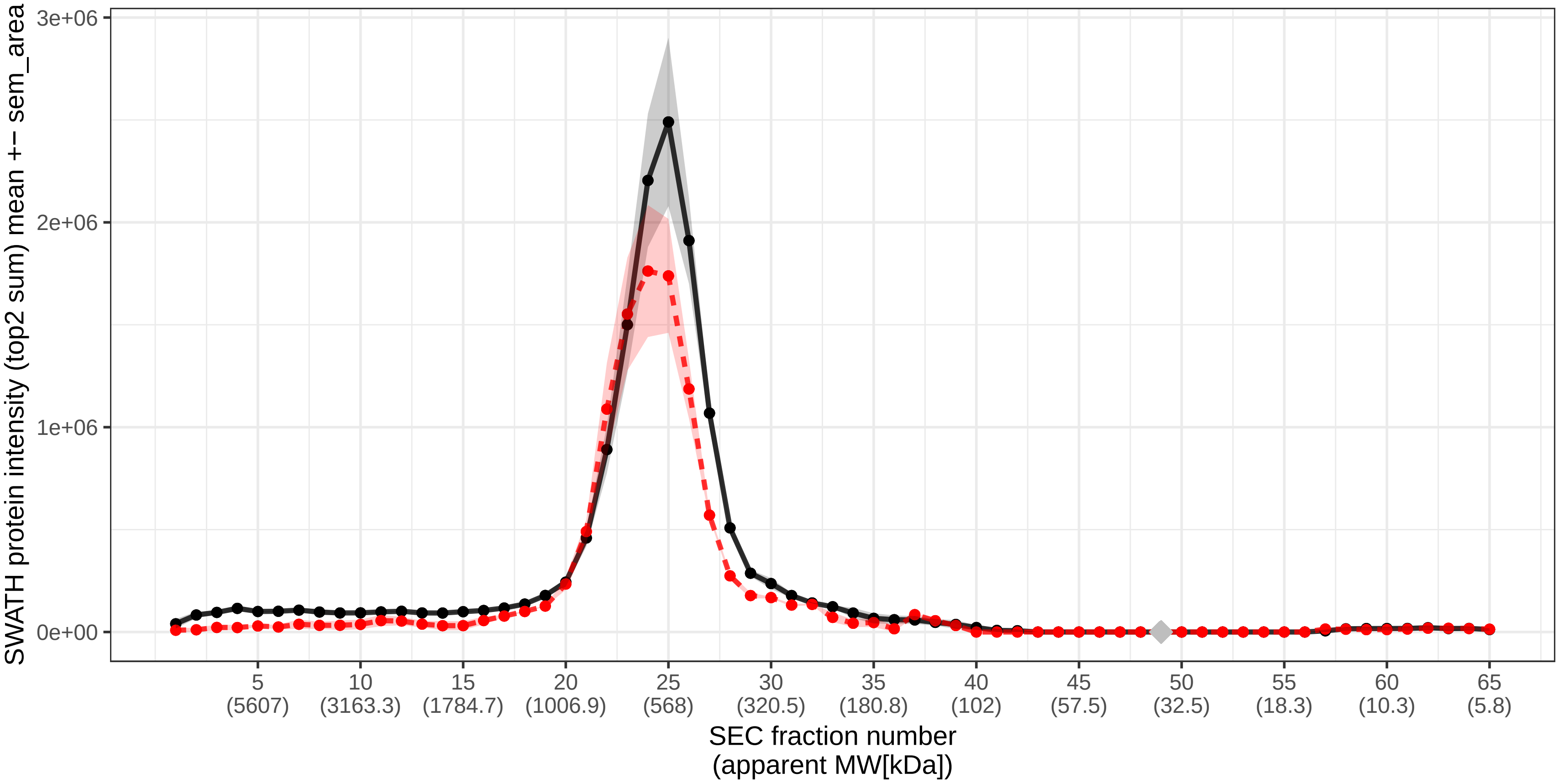

Supplement: Data S1. SEC-SWATH-MS Protein Chromatograms, Related to Figure 1 [file mmc6.zip › SECchrom_Q9BQA1_MEP50_HUMAN_WDR77_MEP50_WD45_HKMT1069_Nbla1.pdf]
